# Reducing and Improving Patient Observations in a London Psychiatric Ward: A Quality Improvement Project

**DOI:** 10.1192/bjo.2026.11370

**Published:** 2026-06-30

**Authors:** Zurima Diaz, Marlon Sesay, Luke Coltman

**Affiliations:** Central and North West London NHS Trust, London, United Kingdom

## Abstract

**Aims:**

Over an 18-month collaborative at CNWL, Thames Ward an acute mental health ward at St Charles Hospital in the Royal Borough of Kensington and Chelsea, aimed to reduce high-level observations by 50% and decrease the frequency of continuous observation. Baseline data showed 18 high-level observations per week and use of continuous observation every 2.5 days.

**Methods:**

With support from an Improvement Coach and workshops, the team tested three PDSA-cycle changes: safety huddles including all staff in observation decisions, prioritising risk assessments over automatic observation for new admissions, and improving patientcommunication. Seven in-depth interviews by an Expert by Experience informed communication practices.

**Results:**

The project achieved a 50% reduction in high-level observations (18 instances down to 9) sustained from January–September 2025. Continuous observation frequency decreased, with up to 43 days between episodes. Violence and aggression incidents did not increase, and bank staff costs fell to £0 in September 2025.

**Conclusion:**

This work demonstrates that restrictive practices can be reduced without compromising safety, highlights the importance of challenging entrenched routines, and shows that Expert by Experience involvement is central to meaningful improvement.

